# Supplementary material for: Bayesian probabilistic projections of proportions with limited data: An application to subnational contraceptive method supply shares
Source: PLoS One. 2026 Apr 24;21(4):e0345413. doi: 10.1371/journal.pone.0345413 (PMC13108902; doi:10.1371/journal.pone.0345413)
Supplement: S1 File — (PDF) [file pone.0345413.s001.pdf]

Supplementary Materials: Bayesian probabilistic projections of proportions with limited data

Hannah Comiskey[1*], Niamh Cahill [2], Leontine Alkema [3], David T. Frazier [1], Worapree Maneesoonthorn[1].

1 Department of Econometrics and Business Statistics, Monash University, Melbourne, Australia.
2 Department of Mathematics and Statistics, Maynooth University, Kildare, Ireland.
3 Department of Biostatistics and Epidemiology, University of Massachusetts Amherst,Amherst, MA, USA.

* hannah.comiskey@monash.edu

Subnational administration model estimates

The complete set of subnational supply share estimates for every country and subnational administration region can be reproduced using the code and data found on the github, `https://github.com/hannahcomiskey/Comiskey_PlosOnepaper`. The DOI of the dataset is DOI: 10.18128/D080.V11 (2024).

Data imputation

In some instances, the calculated standard error was 0, where a particular method came entirely from one sector. In these cases, we imputed the standard error of these observations using a binomial distribution approximation of the variance, with consideration given to the design effect (DEFT) of the individual DHS surveys [48]. For additional caution, we did not impute the standard errors for observations where the sample size was less than 20 women. In such cases, observations requiring standard error imputation with sample sizes below 20 women were removed from the dataset. This procedure resulted in 72 observations in the method–supply share database receiving imputed standard errors.
A database of DHS survey design effects for a sample of countries and surveys was collected for illustration purposes. The DHS DEFT values can be found in the final DHS report, Appendix B, "Estimates of Sampling Errors" for each DHS survey in a given country and year [49]. The DHS survey design effect measures the impact of the complex DHS sampling design on the variance of a given estimator, in this case, the contraceptive method supply shares in a given country. DEFT values represent the ratio of the variance of a parameter estimated under a complex sampling design to that obtained under simple random sampling [50]. In this context, the DEFT captures the inflation (DEFT >1) or deflation (DEFT <1) of our imputed standard errors due to the complexity of the DHS sampling process. The imputed standard error is given by:

$$\widehat{SE}_{p,t} = \sqrt{\frac{\widehat{v}(1-\widehat{v})}{N_1+1}} \times \text{DEFT}_{p[c],t},$$

where:

- $\widehat{SE}_{p,t}$ is the imputed standard error for province p at year t;
- $\text{DEFT}_{c[p],t}$ is the DHS survey design effect for province p in country c at year t; if $\text{DEFT}_{c[p],t}$ is not available in the database, it is set to 1.5, the average of the observed DEFT values;
- N_1 is the number of observations in the data where the observed proportion exceeds 0.99;
- $\widehat{\upsilon} = \dfrac{N_1 + 1}{N_{\text{all}} + 1}$;
- N_{all} is the total number of observations in the data.

Out of sample validation

Metrics

To validate our model, we split our data into test and training sets. The observed survey data from 1990 to 2014 inclusive was considered the training set, while any data beyond 2015 was labeled as the test set and withheld for validation. Using the training set, we estimate a predictive distribution of the method supply shares supplied by the public sector for each method and subnational administration region in our training dataset from 1990 to 2024. We then compared the resulting predictive distributions with the observed public sector test set data. The test set contained 16 countries, covering 81 subnational administration regions and all five contraceptive methods. In total, the test set contained 338 observations while the training set contains 931 observations. We evaluated the results of the validation using different measures of accuracy and prediction interval calibration.

To assess the accuracy of our mean point predictions, we consider the mean absolute relative error (MARE) in percentage points. The MARE is negatively orientated, therefore smaller MARE values are better. The MARE is given by:

$$MARE = 100 \times \frac{1}{PTM} \sum_{p,t,m} \frac{|y_{p,t,m} - \hat{y}_{p,t,m}|}{\hat{y}_{p,t,m}} \tag{1}$$

where, $\mathrm{y_{p,t,m}}$ is the observed proportion of method m, at time t, in subnational administration region p supplied by the public sector. $\mathrm{y_{p,t,m}}$ is part of the test set. $\hat{y}_{p,t,m}$ is the corresponding estimated proportion supplied by the public sector. P is the number of subnational administration regions in the test set. T is the number of time periods in the test set. M is the number of contraceptive methods in the test set.

The standardized absolute prediction error (SAPE) is a measure of the dispersion of the generated predictive distributions. Ideally, we expect the SAPE value to be 1. When the values observed in the test set are more spread out than the predictive distributions, we say that the distribution is under-dispersed. In these instances the SAPE value will be greater than 1. Conversely, when the predictive distribution is over-dispersed, the SAPE value will be less than 1. The SAPE is given by:

$$SAPE = 1.4826 \times \text{median}(\frac{|y_{p,t,m} - \hat{y}_{p,t,m}|}{\hat{\sigma}_{p,t,m}}) \tag{2}$$

where $\hat{\sigma}_{p,t,m}$ is the estimated Bayesian predictive standard deviation associated with the test set observation $\mathrm{y_{p,t,m}}$.

We calculate error terms, $\mathrm{e_{p,t,m}}$, to describe the difference between the test set public sector survey observation, $\mathrm{y_{p,t,m}}$, and the corresponding mean estimate from the posterior predictive distribution, $\hat{y}_{p,t,m}$ such that,

$$e_{p,t,m} = y_{p,t,m} - \hat{y}_{p,t,m}. \tag{3}$$

To evaluate the accuracy of our model, we considered the root mean square error (RMSE) of the public sector test set estimates. Let,

$$\text{RMSE} = \sqrt{\frac{\sum_{p,t,m} e^2_{p,t,m}}{N}}, \tag{4}$$

where, N is the number of observations in the test set. $\mathrm{e_{p,t,m}}$ is the error calculated for the proportion of method m, at time t, in subnational administration region p supplied by the public sector. The RMSE can be interpreted as the average error observed across all subnational administration regions, time points and methods in the test set.

Coverage assumes that if our model is correctly calibrated, then for each sector the model should be able to capture the test set of out-of-sample observations with 80% and 95% accuracy. To examine the bias of our models estimates, we examined the location of the incorrectly estimated test set observations for the 95% coverage instance. We expect that the 5% of observatsions are incorrectly estimated. These incorrectly estimated observations should approximately evenly distributed above and below the estimated 95% prediction interval. By examining the breakdown of location, we are evaluating the tendency of the bias of our model, i.e. whether the model tends to under- or over-estimate the test set. A larger proportion of observations located below the prediction interval, shows that the model is tending to over-estimate the test set. Conversely, when a higher proportion of the incorrectly estimated observations are located above the prediction interval, the model tends to under-estimate the test set. Finally, we also consider the median estimate of the 95% predictive interval widths for each test set observation across all subnational administration regions, time points and methods.

Modelling alternatives

To justify the complexity of our proposed model, the 'multivariate intercept P-spline model', we compared it against suitable alternatives. To begin, we compared the proposed model against the model described in Comiskey et al. (2024). We refer to this model as the 'multivariate delta' model. We ran the multivariate delta model at the subnational level, including estimated subnational correlations across methods for the rates of change in spline coefficients, for the public/private sector breakdown of method supply shares over time. We also took the 0-covariance model from this paper to include as a baseline model. In this instance, we refer to the 0-covariance model of Comiskey et al. 2024 as the '0-covariance P-spline' model. For complete descriptions of these models, please refer to the aforementioned paper.

To evaluate the impact of estimating our spline coefficients using the forward/backward approach, we also include B-spline models with spline coefficients estimated using a traditional AR(1) process.

To begin, we consider a hierarchical Bayesian model that combines basis splines for smooth predictions over time, with method-, subnational administration region-specific

intercepts and shrinkage triple gamma priors for regularization [51]. We will refer to the model as the 'shrinkage P-spline' model. As with our proposed model, we set a sum to zero constraint on the spline coefficients to ensure identifiability. The B-spline model links the observed data to the model's predictions using a logit-normal data model, informed by the observed logit-transformed standard errors of the survey data. For the public sector component of interest, $\phi_{p,t,m,1}$, the B-spline model is set up as follows:

$$\text{logit}\left(\phi_{p,t,m,1}\right) = \psi_{p,t,m} = \alpha_{p,m} + \sum_{k=1}^{K} \beta_{p,m,k} B_k(t) \tag{5}$$

Where, the intercept term, $\alpha_{p,m}$, is estimated hierarchically such that,

$$\alpha^{country}_{c,m} \mid \sigma_{\alpha^{country}_m} \sim N(0, \sigma^2_{\alpha^{country}_m}), \tag{6}$$

$$\alpha^{prov.}_{p,m} \mid \alpha^{country}_{c[p],m}, \sigma_{\alpha^{prov.}_m} \sim N(\alpha^{country}_{c[p],m}, \sigma^2_{\alpha^{prov.}_m}). \tag{7}$$

The standard deviation terms of the hierarchically estimated intercept terms are given vague truncated Cauchy and Normal priors. Such that,

$$\sigma_{\alpha^{country}_m} \sim C^{+}(0, 1), \tag{8}$$

$$\sigma_{\alpha^{prov.}_m} \sim N^{+}(0, 2^2). \tag{9}$$

To estimate the spline coefficients, we use an auto-regressive process such that the expected value of each spline coefficient is that of the previous spline coefficient. Included in this estimation process is a triple gamma shrinkage prior. This prior shrinks the effects of a given spline coefficient towards 0 when it is not considered a significant contributor [51]. Regularizing the spline coefficients via a shrinkage prior reduces the possibility of over-fitting. For this model, we used the default parameter settings as used in the shrinkTVP R package [52].

$$\beta_{p,m,k} \mid \beta_{p,m,k-1}, \sigma_{\beta_m} \sim N(\beta_{p,m,k-1}, \sigma^2_{\beta_m}) \tag{10}$$

$$\sigma_{\beta_m} \mid \xi^2_m \sim N^{+}(0, \xi^2_m) \tag{11}$$

$$\xi^2_m \mid a^{\xi}, \kappa^2_m \sim Gamma(a^{\xi}, \frac{a^{\xi}\kappa^2_m}{2}) \tag{12}$$

$$\kappa^2_m \mid c^{\xi}, \kappa^2_B \sim Gamma(c^{\xi}, \frac{c^{\xi}}{\kappa^2_B}) \tag{13}$$

$$2a^{\xi} \sim Beta(5, 10) \tag{14}$$

$$2c^{\xi} \sim Beta(5, 2) \tag{15}$$

$$\frac{\kappa^2_B}{2} \sim F(1, 1) \tag{16}$$

In addition this B-spline model, we also considered a multivariate normal B-spline. We begin using the same formation for $\psi_{p,t,m}$ as given in equation 5. In this model, we estimate the spline coefficients and intercept parameters using multivariate Normal priors. The estimation of the intercept $\alpha^{prov.}_{p,m}$ uses the same hierarchical multivariate normal priors as described in equations 5 to 7 of the main paper. For the estimation of the spline coefficients, we use a multivariate Normal prior such that,

$$\beta_{p,1:M,k} \mid \beta_{p,1:M,k-1}, \Sigma_{\beta} \sim MVN(\beta_{p,1:M,k-1}, \Sigma_{\beta}). \tag{17}$$

As before, we use a sum-to-zero constraint on the spline coefficients to ensure idenitifability and a vague Wishart prior to estimate the variance-covariance matrix of the spline coefficients:

$$\Sigma_{\beta} \sim Wishart(I_M, M + 1). \tag{18}$$

Prior sensitivity analysis

To assess the robustness of our estimated correlations, we conducted prior sensitivity checks by increasing the degrees of freedom in the Inverse-Wishart prior used for the variance–covariance matrices of the spline coefficients. Specifically, we considered a more informative prior of the form

$$\Sigma_\theta \sim IW(I_M, M+5),\ \Sigma_\alpha \sim IW(I_M, M+5), \tag{19}$$

where the additional degrees of freedom impose stronger shrinkage toward the identity matrix. This in turn induces greater shrinkage of the off-diagonal elements, pulling the implied correlations closer to zero. We found no significant difference in the estimated correlations from this more conservative prior.

References

1. FP2030 — United Nations Foundation. What We Do; 2024. Available from: https://www.fp2030.org/about/.

2. FP2030. Family Planning 2020: Rights & Empowerment Principles for Family Planning; 2020. Available from: https://www.fp2030.org/resources/resources-family-planning-2020-rights-empowerment-principles-family-plann

3. DeLong, Rosen R, Holtz J. Understanding Private Sector Domestic Resource Mobilization for Health; 2020.

4. Cahill N, Sonneveldt E, Stover J, Weinberger M, Williamson J, Wei C, et al. Modern contraceptive use, unmet need, and demand satisfied among women of reproductive age who are married or in a union in the focus countries of the Family Planning 2020 initiative: a systematic analysis using the Family Planning Estimation Tool. The Lancet. 2018;391:870–882. doi:10.1016/S0140-6736(17)33104-5.

5. Cutherell A, Doi N, Christofield M, Sathyandran J, Fatima A, Haider H, et al.. Leveraging the Private Sector to Expand Access to Contraception in the Era of Decreasing Donor Funding; 2024. Available from: https://knowledgesuccess.org/2024/06/06/leveraging-the-private-sector-to-expand-access-to-contraception-in-the-er #:~:text=The%20private%20sector%20has%20been,methods%2C%20like%20condoms%20and%20pills.

6. Ross J. Improved Reproductive Health Equity Between the Poor and the Rich: An Analysis of Trends in 46 Low-and Middle-Income Countries. Global Health: Science and Practice. 2015;3.

7. Chakraborty NM, Sprockett A. Use of family planning and child health services in the private sector: An equity analysis of 12 DHS surveys. International Journal for Equity in Health. 2018;17:1–12. doi:10.1186/s12939-018-0763-7.

8. Weinberger M, Callahan S. The Private Sector: Key to Achieving Family Planning 2020 Goals; 2017.

9. World Health Organisation. WHO launches online learning programme to increase access to contraception via pharmacies; 2024. Available from: https://www.who.int/news/item/25-09-2024-who-launches-online-learning-programme-to-increase-access-to-c

10. Corroon M, Kebede E, Spektor G, Speizer I. Key Role of Drug Shops and Pharmacies for Family Planning in Urban Nigeria and Kenya. Global Health: Science and Practice. 2016;4.

11. Bradley SEK, Shiras T, Findings K. Where Women Access Contraception in 36 Low-and Middle-Income Countries and Why It Matters. Global Health: Science and Practice. 2022;1-.

12. Bossert TJ, Beauvais JC. Decentralization of health systems in Ghana, Zambia, Uganda and the Philippines: a comparative analysis of decision space. HEALTH POLICY AND PLANNING. 2002;17:14–31.

13. Wu Y, Li ZR, Mayala BK, Wang H, Gao PA, Paige J, et al.. SPATIAL MODELING FOR SUBNATIONAL ADMINISTRATIVE LEVEL 2 SMALL-AREA ESTIMATION; 2021. Available from: https://dhsprogram.com/pubs/pdf/SAR21/SAR21.pdf.

14. Li Q, Louis TA, Liu L, Wang C, Tsui AO. Subnational estimation of modern contraceptive prevalence in five sub-Saharan African countries: A Bayesian hierarchical approach. BMC Public Health. 2019;19. doi:10.1186/s12889-019-6545-3.

15. Mercer LD, Lu F, Proctor JL. Sub-national levels and trends in contraceptive prevalence, unmet need, and demand for family planning in Nigeria with survey uncertainty. BMC Public Health. 2019;19. doi:10.1186/s12889-019-8043-z.

16. Wakefield J, Fuglstad GA, Riebler A, Godwin J, Wilson K, Clark SJ. Estimating under-five mortality in space and time in a developing world context. Statistical Methods in Medical Research. 2019;28:2614–2634. doi:10.1177/0962280218767988.

17. Ghosh M, Rao JNK. Small Area Estimation: An Appraisal. Statistical Science. 1994;9:55–93. doi:10.1214/ss/1177010647.

18. Fay RE, Herriot RA. Estimates of Income for Small Places: An Application of James-Stein Procedures to Census; 1979.

19. Besag J, York J, Mollii A. BAYESIAN IMAGE RESTORATION, WITH TWO APPLICATIONS IN SPATIAL STATISTICS* **; 1991.

20. Chen C, Wakefield J, Lumely T. The use of sampling weights in Bayesian hierarchical models for small area estimation. Spatial and Spatio-temporal Epidemiology. 2014;11:33–43. doi:10.1016/j.sste.2014.07.002.

21. Alexander M, Zagheni E, Barbieri M. A Flexible Bayesian Model for Estimating Subnational Mortality. Demography. 2017;54(6):2025–2041. doi:10.1007/s13524-017-0618-7.

22. Wah W, Ahern S, Earnest A. A systematic review of Bayesian spatial–temporal models on cancer incidence and mortality; 2020.

23. Tessema ZT, Tesema GA, Ahern S, Earnest A. A Systematic Review of Areal Units and Adjacency Used in Bayesian Spatial and Spatio-Temporal Conditional Autoregressive Models in Health Research; 2023.

24. Peterson EN, Nethery RC, Padellini T, Chen JT, Coull BA, Piel FB, et al. A BAYESIAN HIERARCHICAL SMALL AREA POPULATION MODEL ACCOUNTING FOR DATA SOURCE SPECIFIC METHODOLOGIES FROM AMERICAN COMMUNITY SURVEY, POPULATION ESTIMATES PROGRAM, AND DECENNIAL CENSUS DATA HHS Public Access Author manuscript. Ann Appl Stat. 2024;18:1565–1595. doi:10.1214/23-AOAS1849SUPP.

25. Comiskey H, Alkema L, Cahill N. Estimating the proportion of modern contraceptives supplied by the public and private sectors using a Bayesian hierarchical penalized spline model. Journal of the Royal Statistical Society Series A: Statistics in Society. 2024;doi:10.1093/jrsssa/qnae051.

26. Comiskey H, Cahill N. mcmsupply: An R Package for Estimating Contraceptive Method Market Supply Shares; 2023. Available from: https://arxiv.org/abs/2308.09434.

27. The DHS Program. Antenatal Care; n.d. https://dhsprogram.com/data/Guide-to-DHS-Statistics/Antenatal_Care.htm.

28. Dickson KS, Darteh EKM, Kumi-Kyereme A, Ahinkorah BO. Determinants of choice of skilled antenatal care service providers in Ghana: analysis of demographic and health survey. Maternal Health, Neonatology and Perinatology. 2018;4:14. doi:10.1186/s40748-018-0082-4.

29. The DHS Program. Vaccination; n.d. https://dhsprogram.com/data/Guide-to-DHS-Statistics/Vaccination.htm.

30. Agopian A, Sedrakyan T, Muradyan D, Sahakyan G. The prolonged impact of the COVID-19 pandemic on routine childhood vaccinations in Armenia: a time-series analysis. BMC Public Health. 2025;25:3948. doi:10.1186/s12889-025-24912-5.

31. Hubacher D, Trussell J. A definition of modern contraceptive methods; 2015.

32. Data for Impact (D4I) and United States Agency for International Development (USAID). Source of supply (by method); 2024. Available from: https://www.data4impactproject.org/prh/family-planning/fp/source-of-supply-by-method/.

33. Boyle EH, King M, Sobek M. IPUMS-Demographic and Health Surveys: Version 11 [dataset]. Accessed: 01-09-2024.; 2024. Available from: https://doi.org/10.18128/D080.V11.

34. Smithson M, Verkuilen J. A better lemon squeezer? Maximum-likelihood regression with beta-distributed dependent variables. Psychological Methods. 2006;11(1):54–71. doi:10.1037/1082-989X.11.1.54.

35. Binder DA. On the variances of asymptotically normal estimators from complex surveys. International Statistical Review. 1983;51(3):279–292. doi:10.2307/1402588.

36. Lavrakas PJ. Taylor Series Linearization (TSL). In: Lavrakas PJ, editor. Encyclopedia of Survey Research Methods. Thousand Oaks, CA: SAGE Publications; 2013.

37. Susmann H, Alexander M, Alkema L. Temporal Models for Demographic and Global Health Outcomes in Multiple Populations: Introducing a New Framework to Review and Standardise Documentation of Model Assumptions and Facilitate Model Comparison. International Statistical Review. 2022;90:437–467. doi:10.1111/insr.12491.

38. Track20. Monitoring Progress in Family Planning Estimated Modern Use (EMU): A New Service Statistics-Based Family Planning Indicator; 2020. Available from: http://fpet.track20.org/fpet/.

39. Zhang Z. A Note on Wishart and Inverse Wishart Priors for Covariance Matrix. Journal of Behavioral Data Science. 2021;1. doi:10.35566/jbds/v1n2/p2.

40. Oehlert GW. A note on the delta method. American Statistician. 1992;46:27–29. doi:10.1080/00031305.1992.10475842.

41. Hornik K, Leisch F, Zeileis A, Plummer M. JAGS: A program for analysis of Bayesian graphical models using Gibbs sampling. International Workshop on Distributed Statistical Computing. 2003;doi:10.1002/ana.1067.

42. Plummer M, Stukalov A, Denwood M. Package 'rjags'; 2022. Available from: https://mcmc-jags.sourceforge.io.

43. Vehtari A, Gelman A, Simpson D, Carpenter B, Burkner PC. Rank-Normalization, Folding, and Localization: An Improved (Formula presented) for Assessing Convergence of MCMC (with Discussion)*†. Bayesian Analysis. 2021;16:667–718. doi:10.1214/20-BA1221.

44. Shah NM, Wang W, Bishai DM. Comparing private sector family planning services to government and NGO services in Ethiopia and Pakistan: How do social franchises compare across quality, equity and cost? Health Policy and Planning. 2011;26. doi:10.1093/heapol/czr027.

45. White JN, Corker J. Applying a Total Market Lens: Increased IUD Service Delivery Through Complementary Public-and Private-Sector Interventions in 4 Countries; 2016. Available from: www.ghspjournal.org.

46. Elnakib S, Elsallab M, Wanis MA, Elshiwy S, Krishnapalan NP, Naja NA. Understanding the impacts of child marriage on the health and well-being of adolescent girls and young women residing in urban areas in Egypt. Reproductive Health. 2022;19. doi:10.1186/s12978-021-01315-4.

47. Moazzam A, Farron M. Ensuring contraceptive security through effective supply chains; 2017. Available from: https://www.who.int/publications/i/item/WHO-RHR-17.09.

48. ICF International. Demographic and Health Survey Sampling and Household Listing Manual; 2012.

49. Demographic and Health Surveys. Final DHS Reports, Appendix B: Estimates of Sampling Errors; n.d.

50. Park I, Lee H. Variance Estimation Using Taylor Linearization in Complex Sample Surveys. Survey Research. 2004;5(1):25–44.

51. Cadonna A, Frühwirth-Schnatter S, Knaus P. Triple the gamma—a unifying shrinkage prior for variance and variable selection in sparse state space and TVP models. Econometrics. 2020;8. doi:10.3390/econometrics8020020.

52. Knaus P, Bitto-Nemling A, Cadonna A, Frühwirth-Schnatter S. Shrinkage in the Time-Varying Parameter Model Framework Using the R Package shrinkTVP. Journal of Statistical Software. 2021;100. doi:10.18637/JSS.V100.I13.
